# A Rare Presentation of Anticholinergic Toxicity in a Young Patient Due to Over-The-Counter Cold Medicines

**DOI:** 10.7759/cureus.13919

**Published:** 2021-03-16

**Authors:** Ahsum Khan, Gagan Singh, Jason Jacob

**Affiliations:** 1 Internal Medicine, University of Connecticut School of Medicine, Farmington, USA; 2 Internal Medicine, Hartford Hospital, Hartford, USA

**Keywords:** anticholinergic overdose, anticholingeric delerium, altered mental state, otc medication

## Abstract

Anticholinergic toxicity is a relatively uncommon occurrence. When it does occur, it is usually attributed to an overdose of anticholinergic agents, especially in the elderly population. In younger patients, anticholingeric toxicity is usually due to an intentional overdose in a suicide attempt, accidental exposure to jimson weed or deadly nightshade plant, or the combination of anticholinergic drugs with recreational drugs (psilocybin mushroom).

Over-the-counter cold medicines are well known to contain a variety of anticholinergic substances, the most well-known being diphenhydramine. Uncommonly, these readily available substances can produce anticholinergic toxicity in elderly patients, even when appropriate dosages are consumed. Younger patients are less likely to develop this clinical picture, due to higher renal clearance and lower drug volume of distribution. Nonetheless, clinical suspicion should remain high in the younger population. Patients can present with fever, tachycardia, diplopia, urinary retention, dry mucous membranes, and altered mental status. This case provides awareness to the unlikely complication of over-the-counter cold medicines in a young 19-year-old patient, while highlighting the need for diligent history taking and deliberate use of physostigmine in patients with altered mental status.

## Introduction

Anticholinergic toxicity is usually attributed to an overdose of anticholinergic agents, although mild toxicity can be noted as a side effect of these drugs. Most of these anticholinergic agents are ingested by mouth, such as atropine, scopolamine, jimson weed, tricyclic antidepressants (TCAs), and overactive bladder antimuscarinics (oxybutynin, tolterodine, and solifenacin) [1]. Importantly, the anticholinergic effects of these medications are synergistic, meaning the use of multiple agents results in a greater risk for anticholinergic toxicity [1,2].

In terms of pathophysiology, anticholinergic agents compete with acetylcholine at the postganglionic parasympathetic muscarinic receptors. Muscarinic receptors can be found in the salivary glands, smooth muscle, heart, eye, and central nervous system. As a result, during anticholinergic toxicity, patients can present with anhidrosis, tachycardia, fever, altered mental status (confusion, hallucinations, agitation, or delirium), urinary retention, mydriasis, diplopia, and dry mucous membranes [2,3]. In particular, toxicity due to diphenhydramine can present with cardiovascular findings, due to binding with calcium and sodium channels, leading to prolonged QT and QRS complexes [1,2].

In 2018, the American Association of Poison Control Centers reported 3613 single exposures to anticholinergics, 74,698 single exposures to antihistamines, with 30,883 specific to diphenhydramine [4,5]. And of these exposures, diphenhydramine was responsible for 22 out of 24 deaths attributable to antihistamines. In 2017, the sales of cold medications totaled over 150 million units accounting for almost 900 million dollars in sales [4,5]. These medications commonly contain antihistamines, particularly diphenhydramine, which can be an unrecognized cause of delirium in patients.

In this report, we describe the case of a 19-year-old male with a past medical history of asthma who presented to the emergency department (ED) with altered mental status and slurring of speech in the context of frequent intake of over-the-counter (OTC) cold medicine due to an upper respiratory infection. He was found to be tachycardic to 110 beats per min, hypertensive to 160/110 mmHg, with a prolonged QTc of 545 ms, and responded with a rapid improvement in mental status, following a dose of physostigmine for suspected anticholinergic toxicity.

Presentation of anticholinergic toxicity in the general population can vary greatly. Although it is more commonly seen in the elderly, due to decreased renal clearance, and body fat composition, younger patients can also be affected [2,3]. In this younger cohort, toxicity can commonly present as an intentional suicide attempt, accidental ingestion of drugs or plants (jimson weed (*Datura stramonium*) or deadly nightshade (*Atropa belladonna*)) by children, or combination of anticholinergic medicines and recreational drugs (psilocybin mushrooms) [1,3]. However, an important, often overlooked cause in both young and elderly populations is the use of OTC cold medications. Diligence during history taking can be critical, in teasing out the intake of multiple cold medications, quantity, and frequency. 

## Case presentation

A 19-year-old male with a past medical history of asthma presented to the ED with suspicion for altered mental status due to slurred speech and decreased alertness. He was completely alert and oriented the evening prior to presentation, but was complaining of increased fatigue and viral-like symptoms (cough, congestion, and rhinorrhea) that had been present for over six days.

At 3 AM, his mother heard him walking around his room and subsequently heard a loud noise. She quickly went to investigate the noise, and found her son confused, lying face down in bed, and slurring his speech. Emergency Medical Service was called, and he was found to be hypertensive to 160/110 mmHg, tachycardic to 110 beats per minute, and his fasting glucose was found to be 49 mg/dl. A bolus of D50 was given, but produced no positive impact on his clinical state. Salicylate, ammonia, acetaminophen, and thyroid hormone levels were all within normal limits. A complete metabolic panel, complete blood count, venous blood gas, carboxyhemoglobin, urine toxicology, CT scan of the head, chest x-ray, and lumbar puncture were all checked, all of which were non-significant. An EKG revealed sinus tachycardia, with prolonged QTc to 545 ms. Patient was also found to be hypertensive. After these investigations yielded no results, a more detailed physical and history was obtained.

The patient’s mother stated that the patient had been ingesting the OTC cold medications Nyquil and Simply Sleep, both containing diphenhydramine, quite frequently. She also noted that the patient had taken a full 16 oz bottle of Nyquil, over the course of 6 hours preceding the hospital presentation. Closer physical exam revealed 6 mm dilated pupils, flushing of his skin, altered mental status, all consistent with anticholinergic toxicity. In the context of the patient’s presentation, 2 mg of physostigmine was given over 5 min, and the patient became fully alert and oriented within 30 min. He was monitored in the intensive care unit, but did not require further physostigmine. He was discharged with close follow-up the next day.

## Discussion

This case describes an unusual complication of OTC cold medicine usage in a relatively young patient. Anticholinergic delirium is a relatively rare phenomenon in the younger population. Younger individuals tend to have a greater resilience to the anticholinergic toxicity, so accidental overdose on OTC cold medications is rare [6,7]. Our patient’s combination of Simply Sleep and NyQuil introduced the H1 antagonists, doxylamine and diphenhydramine, into his system. Together, even in relatively low doses, these drugs can produce a dramatic anticholinergic effect [1]. It was evident that this patient had not been taking the appropriate doses of these medications, which led to the enhanced depression of his muscarinic activity, and the corresponding clinical picture.

In regard to clinical picture, classical anticholinergic toxicity can be remembered using the phrase “red as a beet, hot as a hare, dry as a bone, mad as a hatter, blind as a bat, and full as a flask” [1]. Therefore, the typical patient will present with flushing, fever, dry mucous membranes, altered mental status/delirium, mydriasis, diplopia, and urinary retention. Rarely, patients can present with seizures, jerking movements, and rhabdomyolysis [1]. As aforementioned, diphenhydramine has been associated with QT and QTC prolongation due to action on sodium and calcium channels [1,2].

Diagnosis of anticholinergic toxicity should follow a systematic approach, one that is consistent with workup of any patient suspected poisoning. The patient’s airway, breathing, and circulation should all be initially assessed. Vital signs should be obtained and evaluated, including heart rate, respiratory rate, pulse oximetry, and blood pressure. The patient should be placed on continuous cardiac monitoring, and have intravenous (IV) access in place [1,2]. Diagnostic testing, including liver panel, urine toxicology screen, creatine kinase, basic metabolic panel, salicylate and acetaminophen level, should all be obtained. The diagnosis is largely based upon clinical findings and history of presentation, as other diagnoses are excluded as mentioned above [2,3].

Management of this presentation is largely supportive. However, in cases where presentation is severe, or signs of altered mental status are observed (central nervous system effects), as was the case in our patient, physostigmine can be used. Physostigmine is an acetylcholinesterase inhibitor that demonstrates peripheral central nervous system activity. The recommended dose is 0.5 to 2 mg IV for adults [1,3]. If symptoms do not respond within 30 min, a second dose may be warranted. In cases of severe diphenhydramine poisoning that does not respond to physostigmine, IV fat emulsion therapy has been proven to be useful. If the patient demonstrates seizure activity, benzodiazepines and correcting electrolyte disturbances may be indicated. Activated charcoal can be used, if indigestion of the anticholinergic drug is within 1 h of presentation. Finally, the hyperthermia in anticholinergic poisoning is classified as an anhidrotic hyperthermia. Sweat glands are innervated by muscarinic receptors [1]. Blockade of these receptors by anticholinergic medications leads to interference with normal heat dissipation methods (sweating), potentially leading to hyperthermia. Hyperthermia in anticholinergic poisoning should be treated with active cooling measures, as antipyretics will not be helpful as this fever is not cytokine based (as opposed to infectious etiology of fever) [1,2,6]. Additionally, atypical antipsychotics should be used with caution, when dealing with encephalopathy due to the risk of hyperthermia and prolonged QTc. A TCA level should be checked in the urine prior to giving physostigmine, due to risk of asystole [2,3].

## Conclusions

This report described the case of anticholinergic toxicity secondary to OTC cold medicine usage in a relatively young patient. Outright delirium is rare in young patients secondary to OTC cold medicine usage, due to increased resilience. This resilience stems from a combination of higher renal clearance of drugs, less volume of distribution, and increased brain reserve. However, it is still possible, and clinical suspicion is still warranted in these cases. Management of severe cases may include the use of physostigmine. If seizures are present, correcting electrolyte disturbances and abortive therapy with benzodiazepines are indicated. With decisive clinical judgment, quick disbursement of acetylcholinesterase inhibitor medication, and supportive measures, prognosis is good overall.
